# Fungal diversity in grape must and wine fermentation assessed by massive sequencing, quantitative PCR and DGGE

**DOI:** 10.3389/fmicb.2015.01156

**Published:** 2015-10-23

**Authors:** Chunxiao Wang, David García-Fernández, Albert Mas, Braulio Esteve-Zarzoso

**Affiliations:** Departament de Bioquímica i Biotecnologia, Facultat d'Enologia, Universitat Rovira i VirgiliTarragona, Spain

**Keywords:** culture-independent techniques, pyrosequencing, SO_2_ treatment, community diversity and composition, wine yeast

## Abstract

The diversity of fungi in grape must and during wine fermentation was investigated in this study by culture-dependent and culture-independent techniques. Carignan and Grenache grapes were harvested from three vineyards in the Priorat region (Spain) in 2012, and nine samples were selected from the grape must after crushing and during wine fermentation. From culture-dependent techniques, 362 isolates were randomly selected and identified by 5.8S-ITS-RFLP and 26S-D1/D2 sequencing. Meanwhile, genomic DNA was extracted directly from the nine samples and analyzed by qPCR, DGGE and massive sequencing. The results indicated that grape must after crushing harbored a high species richness of fungi with *Aspergillus tubingensis, Aureobasidium pullulans*, or *Starmerella bacillaris* as the dominant species. As fermentation proceeded, the species richness decreased, and yeasts such as *Hanseniaspora uvarum, Starmerella bacillaris* and *Saccharomyces cerevisiae* successively occupied the must samples. The “*terroir*” characteristics of the fungus population are more related to the location of the vineyard than to grape variety. Sulfur dioxide treatment caused a low effect on yeast diversity by similarity analysis. Because of the existence of large population of fungi on grape berries, massive sequencing was more appropriate to understand the fungal community in grape must after crushing than the other techniques used in this study. Suitable target sequences and databases were necessary for accurate evaluation of the community and the identification of species by the 454 pyrosequencing of amplicons.

## Introduction

Investigating the fungal community in grape must and wine fermentation is relevant for understanding its relationship with the grape sanitary status and the final wine characteristics (Bokulich et al., 2014). Recently, the development of next-generation sequencing provided a useful tool for the description of prokaryotic and eukaryotic microbial communities that exist in grape leaves, berries, must and wineries (Bokulich et al., 2013, 2014; David et al., 2014; Pinto et al., 2014; Taylor et al., 2014; Valera et al., 2015). The common approach used in these studies was targeted metasequencing: generic target sequences were amplified by PCR to establish a library; then amplicons were sequenced; and identification was performed by comparison with known sequences in databases (Huggett et al., 2013; Mayo et al., 2014). These studies indicated advances relative to the traditional culture-dependent techniques: a greater abundance of bacteria and fungi found in grape leaves and berries and higher sensitivity to minor species due to the possibility of massive sequencing in a short time. Moreover, other culture-independent techniques have played important roles in monitoring the main yeast dynamics during wine fermentation for the last 10 years (Mills et al., 2002; Hierro et al., 2006; Andorrà et al., 2010). Thus, the main aim of this study was to apply these techniques to interpret the fungal communities in grape must and wine fermentation from the Priorat region in Spain.

The Priorat region, the second qualified DOC (Denominación de Origen Calificada) wine region in Spain, is located in southwest Catalonia. This region is characterized by its own “*terroir*” (French word widely use in the wine industry and wine marketing that means specific place character): a topsoil of reddish and black slate with small particles of mica, a hot and dry summer climate with different micro-climates due to the hilly landform (average annual rainfall is 400–600 mm), and vineyards on terraced slopes at altitudes between 100 and 700 m above sea level (Robinson, 2006; Hudin and Serra, 2013). However, few studies have reported on the native microbial ecology of grapes in this region. Torija et al. (2001) investigated the yeast population in spontaneous fermentation from this region over 3 years and reported a unique ecology of yeast species and *Saccharomyces* strains. To investigate the probable fungal “*terroir*” of this region, grapes from slopes at altitudes 400 m above sea level in the villages of Poboleda, Escaladei, and Porrera were crushed into must and fermented in this study. The fungal diversity from grape must and fermentation samples was analyzed by culture-dependent techniques and culture-independent techniques and compared among different samples. The effect of SO_2_ treatment on fungal diversity was also evaluated by low-dosage addition to two grape must varieties from Porrera.

## Materials and methods

### Spontaneous fermentations

Mature grapes (Carignan and Grenache) were randomly taken from vineyards in three villages (Poboleda, Escaladei, and Porrera) of the Priorat region (Spain) in 2012. The grapes were hand-harvested from the plants with gloves and kept in sterile bags in an ice box for transportation. Approximately 1.8 L of grape must was obtained from each 2 kg of grapes at different locations, which were crushed sterilely in the same plastic bag by hand and put into 2 L bottles for spontaneous fermentation. The fermentations were performed at 24°C with 120 rpm agitation speed, and 30 ppm of SO_2_ was added at 24 h in the form of potassium metabisulfite. The fermentation proceeded in semianaerobic conditions as the bottles are not tightly closed and some gas exchange is allowed. All the fermentations were monitored daily using a Densito 30PX Portable Density Meter (Mettler Toledo, Spain), and samples were taken at five different fermentation stages: 0 h (grape must after crush), 24 h (before SO_2_ treatment), 48 h (24 h after SO_2_ treatment), middle stage (density approximately 1040–1060 g/L) and end stage (stable density less than 1000 g/L). Fresh samples were directly analyzed by culture-dependent techniques; cell pellets from 1 mL of samples at each fermentation stage were collected by centrifugation after washing with sterile water and kept at −20°C for further culture-independent analysis by qPCR, DGGE, and massive sequencing techniques.

### Culture-dependent techniques

One milliliter of sample at each fermentation stage was diluted in series and spread onto YPD med/ium (2% glucose, 2% peptone, 1% yeast extract and 1.7% agar) and Lysine medium (Oxoid, USA) for incubation at 25°C for 2–3 days. For plating, a Whitley Automatic Spiral Plater (AES Laboratoire, France) was used, and the viable yeast quantification was performed using a ProtoColHr automatic colony counter (Microbiology International, USA). For further colony identification, 25 colonies were selected randomly from YPD and Lysine plates of each sample (50 colonies in total for each sample) and identified by 5.8S-ITS-RFLP analysis and 26S rDNA D1/D2 domain sequencing. In 5.8S-ITS-RFLP analysis, colony amplifications were first performed by primer pairs of ITS1/ITS4 as described by Esteve-Zarzoso et al. (1999). The amplification products were digested by five restriction enzymes (*Hinf* I, *Hae*III, *Cfo*I, *Dde*I, and *Mbo*I), and corresponding restriction profiles were identified according to Esteve-Zarzoso et al. (1999) and Csoma and Sipiczki (2008). Then, 26S rDNA D1/D2 domain sequencing was used to confirm the colony identification. Each PCR reaction was performed with primer pairs of NL1/NL4 and the program described by Kurtzman and Robnett (1998). An ABI3730 XL DNA sequencer (Macrogen, Korea) was used for the sequencing process, and corresponding sequence alignment was performed by BLAST from the NCBI database (http://blast.ncbi.nlm.nih.gov/).

### DNA extraction

DNA was extracted from the cell pellets stored at −20°C using the DNeasy Plant minikit (Qiagen, USA) as described in Hierro et al. (2006). The same extraction protocol was used for DGGE, qPCR and massive sequencing analyses.

### DGGE analysis

The PCR reactions were performed using a Gene Amp PCR System 2720 (Applied Biosystems, USA) with Primers U1^GC^ and U2 (Meroth et al., 2003). The DGGE procedures followed the description in Andorrà et al. (2008) with a modified DGGE gel using a denaturing gradient from 35 to 55% urea and formamide.

### qPCR analysis

The qPCR reactions were performed using an Applied Biosystems 7300 Fast Real-Time PCR System (Applied Biosystems, USA) with primers for total yeast, *Saccharomyces, Hanseniaspora*, and *Starmerella bacillaris* as described in Andorrà et al. (2010). Standard curves were built for each yeast species in triplicate using 10-fold serial dilutions of fresh cultures.

### Massive sequencing analysis

A fragment of approximately 600 nt from D1/D2 of 26S rDNA was amplified using modified NL1/NL4 primers, which were designed with adaptor and molecular identifier (MID) sequences specially for massive sequencing (Invitrogen, USA). The whole sequencing process was performed using a 454 Roche platform with the Genome Sequencing FLX System (LifeSequencing S.L., Spain): DNA libraries with specific MID sequences were built for each sample by target PCR with the improved primers, and then a primer-dimer removal protocol was applied to each PCR product to increase the sequencing throughput. An equimolecular pool was generated by quantification of the clean PCR products using the Quan-IT™ PicoGreen® kit (Invitrogen), and sequencing of the pooled samples was performed using a 454 FLX Roche sequencer (LifeScience, USA).

The bioinformatic analysis of each sample was conducted by LifeSequencing S.L. (Spain). Quality control of all sequences was first performed by removing sequences with low quality or length lower than 300 nt and the PCR primers. An updated database of 26S rDNA sequences obtained from GenBank of NCBI was constructed for local alignment comparison. By local alignment comparison, each read was assigned to the most probable operational taxonomic unit (OTU) at different taxonomical levels (family, genera and species) with a confidence cutoff value of 80% and an *e*-value of 10^−5^. Sequences with identity value lower than 80% and *e*-value lower than 10^−5^ were assigned as “no hit.”

The fungal community in each sample was analyzed by different biodiversity and similarity metrics at the species level using Estimate S v9.1.0 (Colwell, 2013). Both Shannon diversity and Simpson diversity were used to evaluate species diversity because Simpson diversity is less sensitive to richness and more sensitive to evenness than Shannon diversity (Colwell, 2009). The estimated species richness was also calculated by a nonparametric estimator, Chao1, which depends on the observed number of singletons and doubletons in a sample. Similarities were evaluated using Jaccard Classic and Bray-Curtis because we focused on comparing community compositions.

## Results

Nine samples were obtained from different stages of fermentations; the details are described in Table 1. They were analyzed by culture-dependent techniques (YPD and Lysine plating) and three different culture-independent techniques (qPCR, DGGE and massive sequencing).

**Table 1:** **Details of nine samples from grape must fermentations**.

**Samples**	**Fermentation stages**	**Grape varieties**	**Locations**	**Coordinates**
I	0 h grape must	Grenache	Poboleda	41.227148, 0.844750
II	0 h grape must	Carignan	Escaladei	41.258156, 0.808214
III	24 h grape must (before SO_2_ treatment)	Carignan	Porrera	41.179651, 0.860334
IV	48 h grape must (24 h after SO_2_ treatment)			
V	0 h grape must	Grenache	Porrera	41.176748, 0.860619
VI	24 h grape must (before SO_2_ adding)			
VII	48 h grape must (24 h after SO_2_ treatment)			
VIII	Middle stage of fermentation (day 3)			
IX	Final stage of fermentation (day 11)			

*The middle and end stages of fermentation were determined by density analysis*.

### Yeast diversity analysis by culture-dependent techniques

The 183 isolates from YPD plates were identified as five different species by 5.8S-ITS-RFLP analysis and 26S-D1/D2 sequencing (Table 2). *Hanseniaspora uvarum* was the most frequently isolated species in all samples except sample IX (the end of fermentation, when *Saccharomyces cerevisiae* dominated). *Starmerella bacillaris* was the second most common species, isolated in samples III, IV, V, VIII, and IX. *Issatchenkia terricola* was mainly isolated from fresh grape must after crushing (sample I, III, V, and VI). *Hanseniaspora valbyensis* and *S. cerevisiae* only appeared in a single sample.

**Table 2:** **The fungal diversity of nine different grape must and fermentation samples evaluated by culture-dependent and culture-independent techniques**.

**Techniques**	**Yeast**	**I**	**II**	**III**	**IV**	**V**	**VI**	**VII**	**VIII**	**IX**
Culture-dependent techniques by YPD plating	Total yeast	^*^	4.80 × 10^3^	^*^	1.51 × 10^8^	3.58 × 10^6^	1.06 × 10^7^	2.10 × 10^7^	3.00 × 10^7^	9.10 × 10^5^
	*Hanseniaspora uvarum*	7/9	^*^	9/25	19/24	14/25	24/25	25/25	22/25	nd
	*Hanseniaspora valbyensis*	nd	^*^	6/25	nd	Nd	nd	nd	nd	nd
	*Issatchenkia terricola*	2/9	^*^	8/25	nd	3/25	1/25	nd	nd	nd
	*Saccharomyces cerevisiae*	nd	^*^	nd	nd	Nd	nd	nd	nd	18/25
	*Starmerella bacillaris*	nd	^*^	2/25	5/24	8/25	nd	nd	3/25	7/25
Culture-dependent techniques by Lysine plating	Total yeast	^*^	2.70 × 10^3^	4.88 × 10^6^	1.41 × 10^7^	1.75 × 10^6^	^*^	1.33 × 10^7^	3.10 × 10^8^	1.10 × 10^5^
	*H. uvarum*	13/25	8/9	25/25	25/25	13/20	^*^	25/25	25/25	nd
	*I. terricola*	9/25	nd	nd	nd	7/20	^*^	nd	nd	nd
	*Starm. bacillaris*	3/25	1/9	nd	nd	Nd	^*^	nd	nd	25/25
qPCR	Total yeast	7.62 × 10^2^	2.85 × 10^5^	6.07 × 10^7^	1.13 × 10^8^	1.82 × 10^4^	6.06 × 10^6^	2.93 × 10^6^	4.35 × 10^5^	2.31 × 10^5^
	*Hanseniaspora*	nd	9.95 × 10^3^	2.46 × 10^7^	1.44 × 10^7^	6.70 × 10^2^	2.64 × 10^6^	3.42 × 10^5^	2.45 × 10^5^	1.94 × 10^4^
	*Saccharomyces*	nd	nd	nd	nd	Nd	nd	nd	nd	5.98 × 10^4^
	*Starm. bacillaris*	nd	nd	1.85 × 10^6^	2.90 × 10^5^	8.93 × 10^2^	1.49 × 10^6^	3.54 × 10^4^	4.67 × 10^2^	nd
DGGE	*Aureobasidium pullulans*	–	+	nd	nd	+	nd	nd	nd	nd
	*Botryosphaeria dothidea*	nd	+	nd	nd	Nd	nd	nd	nd	nd
	*Hanseniaspora opuntiae*	nd	+	nd	nd	+	+	+	+	+
	*H. uvarum*	nd	+	+	+	+	+	+	+	+
	*S. cerevisiae*	nd	nd	nd	nd	Nd	nd	nd	nd	+
	*Starm. bacillaris*	nd	nd	+	+	Nd	+	+	+	+
Massive sequencing	*Aspergillus tubingensis*	55.80%	18.18%	<	<	<	<	–	–	–
	*Aureo. pullulans*	<	18.63%	<	<	<	<	–	–	–
	*B. dothidea*	–	<	–	–	–	–	–	–	–
	*Hanseniaspora thailandica*	–	<	5.25%	5.00%	<	<	<	<	<
	*H. opuntiae*	–	6.05%	<	<	<	<	<	<	–
	*H. uvarum*	–	<	60.78%	56.68%	<	13.57%	11.80%	<	<
	*uncultured Hanseniaspora*	–	<	12.37%	13.44%	<	<	<	<	<
	*I. terricola*	–	–	<	<	<	<	<	<	–
	*Penicillium brevicompactum*	<	5.47%	–	–	<	–	–	–	–
	*Penicillium crustosum*	<	5.56%	–	–	<	–	–	–	–
	*Penicillium glabrum*	8.64%	–	–	–	<	–	–	–	–
	*S. cerevisiae*	–	<	–	–	–	<	–	–	25.98%
	*Uncultured Saccharomyces*	–	<	–	<	–	<	–	–	<
	*Starm. bacillaris*	–	<	17.19%	20.22%	87.86%	79.61%	80.22%	98.10%	71.19%
	*Uncultured soil fungus*	14.45%	<	–	<	<	<	<	–	–

The results from culture-dependent techniques are shown as specie colony numbers compared with total colony numbers, with total yeast concentration also shown (cfu/ml). Grape must samples with molds mainly found on plates, resulting in hard quantification or isolation, are labeled with “ ^*^.” The qPCR results are shown as cells concentration (cells/ml), the species detectable by DGGE are represented by “+,” and the results from massive sequencing are shown as percentages (%). Because of the rich diversity of the massive sequencing results, only the major species with percentages higher than 5% are shown in the table, and the species with lower percentages in some samples, if listed, are marked with “ <.” The symbol of “nd” represents the species with lower concentrations below the detection limit (100 cells/ml) by qPCR and species undetectable by the other three techniques.

The 179 non-*Saccharomyces* isolates from Lysine medium were identified. Only three species were recovered, with *H. uvarum* as the main species (Table 2). *I. terricola* was only isolated from grape must after crushing (sample I and V), and *Starm. bacillaris* was present in grape must after crushing and also at the end of fermentation.

### Yeast population diversity by qPCR analysis

The population levels of total yeast, *Hanseniaspora* spp., *Starm. Bacillaris*, and *Saccharomyces* spp., were separately quantified (Table 2). The total yeast population in grape must after crushing (sample I, II, and V) was lower than 10^6^ cells/mL, but the yeast population then increased to 10^5^ to 10^8^ cells/mL. *Hanseniaspora* was the main genus detected in almost all samples, ranging from 10^2^ to 10^7^ cells/mL. *Starm. bacillaris* mainly appeared in grape must from Porrera (10^2^ to 10^6^ cells/mL), although it was not detected at the end of fermentation. Surprisingly, the *Saccharomyces* population was only detected by this technique at the end of fermentation. The total yeast population size was not affected by the SO_2_ treatment; however, the *Starm. bacillaris* population was reduced by approximately tenfold after SO_2_ addition. This observation was made in the two samples analyzed before and after SO_2_ addition.

### DGGE analysis of grape must samples

The bands obtained in DGGE profiles were assigned to six species by sequencing, as indicated in Table 2. No species were observed from sample I, and in the remaining eight samples, *H. uvarum* appeared in each sample, *Hanseniaspora opuntiae* and *Starm. bacillaris* in six samples, *Aureobasidum* (*Aureo*.) *pullulans* in sample II and V, and *Botryosphaeria dothidea* and *S. cerevisiae* in only one sample.

### Fungal diversity analysis by massive sequencing

#### Species diversity and similarity of grape must samples

A total of 120,081 original sequences were obtained from nine samples, of which 106,095 sequences passed the quality control filter. As shown in Table 3, approximately 10,000 high quality reads were obtained from each sample, and the average sequence length was approximately 500 nt. The similar level of read numbers from each sample established comparability among samples. The analysis of massive sequencing was performed based on taxonomy-dependent methods, by which query sequences were compared with known sequences deposited in annotated databases. After alignment, 247 OTUs were identified at the species level from the 105,541 hit reads, and 554 reads were not assigned an identity in the current eukaryotic database of NCBI (0.5% of no hit reads).

**Table 3:** **Total sequences obtained from massive sequencing and fungal community metrics of all samples**.

**Metrics**	**I**	**II**	**III**	**IV**	**V**	**VI**	**VII**	**VIII**	**IX**
High quality reads	10033	9301	10255	12798	10503	18162	11529	12559	10955
Average length (nt)	499	471	545	539	505	512	510	511	486
Number of OTUs at species level	86	186	22	21	64	25	15	11	15
Number of no hit reads	30	165	55	85	69	61	29	10	50
Estimated species richness	152	329	32	22	96	32	16	11	20
Confidence intervals	113–248	263–451	24–76	21–33	75–153	26–59	15–30	11–17	16–44
Shannon exponential species diversity	5.57	20.61	3.32	3.49	1.99	2.04	1.90	1.12	2.03
Simpson inverse species diversity	2.88	10.94	2.37	2.57	1.28	1.52	1.43	1.04	1.72

*Estimated species richness was calculated using the Chao1 richness estimator, with log-linear 95% confidence intervals. OTU, operational taxonomic unit*.

Rich OTUs were found in the three grape must samples after crushing (I, II, and V). However, the fermentation samples showed a lower OTU richness. The species richness of each sample was estimated by Chao 1, and more OTUs were expected from the three grape must samples after crushing; however, in the other six fermentation samples, the observed OTUs were similar to the estimated species richness. Thus, both observed and estimated species richness decreased as fermentation proceeded, as we expected. The Shannon (exponential form) and Simpson (inverse form) diversity indices were used to evaluate the community diversity, in which both richness and evenness were integrated. The diversity values were no less than 1 due to the corresponding forms used, and higher values meant higher diversity. Thus, sample II presented the highest diversity and the best evenness of the nine samples. Although sample V had a higher value of richness than some fermentation samples (III, IV, VI, IX), its diversity by both indexes was lower, mainly due to its poor evenness.

The community similarity in nine samples was pairwise analyzed using the Jaccard Classic and Bray-Curtis indices (Table 4). Values from both indices range from 0 to 1, with 0 representing no similarity between two samples and 1 meaning no differentiation. Samples I and V showed similarities of 0.271 by Jaccard Classic and 0.066 by Bray-Curtis, which were lower values than the similarities between III and VI (0.516 Jaccard Classic and 0.376 Bray-Curtis) or IV and VII (0.565 Jaccard Classic and 0.377 Bray-Curtis). As noted in Table 1, samples I and V were from the same grape variety (Grenache) but different locations (Poboleda, Porrera), while III/IV and VI/VII were from the same location (Porrera) but from two different grape varieties (Carignan and Grenache). Therefore, the location seemed to contribute more to the dissimilarities between two samples than the grape variety.

**Table 4:** **Community similarity metrics (Jaccard Classic and Bray-Curtis) by pairwise multivariate analysis of all samples (I–IX)**.

		**Bray-Curtis**								
		**I**	**II**	**III**	**IV**	**V**	**VI**	**VII**	**VIII**	**IX**
Jaccard	I		0.357	0.004	0.002	0.066	0.001	0.000	0.000	0.000
Classic	II	0.242		0.087	0.078	0.118	0.062	0.079	0.040	0.045
	III	0.049	0.072		0.887	0.217	0.376	0.343	0.173	0.174
	IV	0.059	0.078	0.593		0.263	0.400	0.377	0.221	0.226
	V	0.271	0.185	0.284	0.288		0.677	0.880	0.822	0.738
	VI	0.078	0.093	0.516	0.704	0.290		0.777	0.819	0.543
	VII	0.020	0.052	0.423	0.565	0.197	0.538		0.809	0.703
	VIII	0.000	0.037	0.375	0.391	0.154	0.333	0.625		0.672
	IX	0.020	0.052	0.276	0.385	0.145	0.379	0.364	0.444	

#### Fungal community composition at different phylogenetic levels

**Figure 1:**
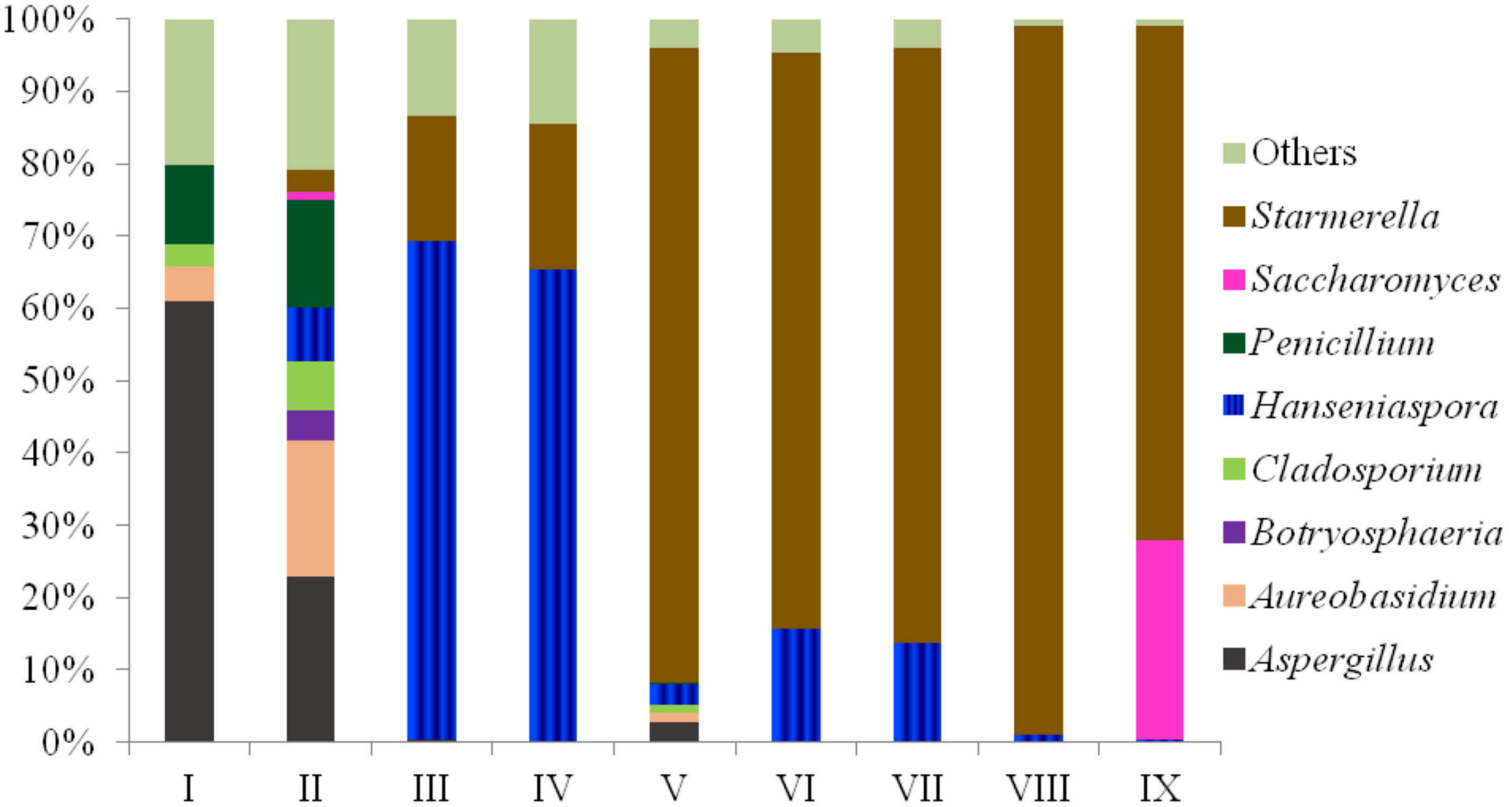
**Community distribution of the eight most abundant genera of nine samples (I–IX) using massive sequencing analysis**.

The fungal communities of the grape must were mainly characterized by high amounts of OTUs from the *Ascomycota* phylum (more than 95% in each sample). Forty-six of the 247 OTUs were present at 0.1–5% in each sample, and 189 OTUs presented a minor proportion (lower than 0.1%). Only 12 species were higher than 5% in each sample, as shown in Table 2. The dominant species were *Aspergillus* (*Asper*.) *tubingensis* in sample I, *Aureo. pullulans* in sample II, *H. uvarum* in samples III and IV, and *Starm. bacillaris* in samples V–IX. Species from the *Eurotiomycetes* and/or *Dothideomycetes* class mainly occupied the grape must after crushing (sample I and II), and most of the species found in grape fermentation must (sample III–IX) were from the *Saccharomycetes* class. At the genus level, the eight most abundant genera in nine samples are listed in Figure 1. Their sum accounts for more than 80% in each sample. The fungal community composition at different phylogenetic levels was more obviously affected by region and grape variety than the SO_2_ treatment, as the latter only caused small percentage changes in some non-*Saccharomyces* species, mainly in the *Hanseniaspora* yeast genus.

### Comparison among culture-dependent techniques and different culture-independent techniques

Comparing the results from different techniques, all the species detected by culture-dependent techniques, qPCR and DGGE were also found by massive sequencing except for sample I; however, the quantity or percentage of some species from the *Hanseniaspora* and *Starmerella* genera varied depending on the techniques used. *Saccharomyces* was found only in sample IX by culture-dependent techniques, qPCR and DGGE, while a minor population was also found in samples II, IV, and VI by massive sequencing. Most of the fungi from the non-*Saccharomycetes* class were detectable by massive sequencing, whereas only dominant species could be found by DGGE. Although they were also observed on YPD or Lysine plates, it was difficult to perform identification and quantification by culture-dependent techniques. Furthermore, non-culturable cells at the end of fermentation, such as *H. uvarum*, were quantifiable or detectable by the three culture-independent techniques.

## Discussion

The nine samples from different locations, grape varieties and corresponding fermentation stages allowed the analysis of yeast diversity and ecology in the Priorat wine region of Spain. However, our study went beyond descriptive analysis and focused on the comparison between culture-dependent techniques and culture-independent techniques to evaluate the fungal diversity based on rDNA-PCR polymorphism. Recent studies have mentioned drawbacks of rDNA-PCR-based methods, especially for culture-independent techniques, such as preferential annealing of the primers, the representativity and quality of DNA, and variable gene copy numbers in different species, and these drawbacks might lead to overestimation/underestimation of the proportion of some species in the overall fungal community (Andorrà et al., 2008; Angly et al., 2014; Valera et al., 2015). Although, it was also observed in this study that massive sequencing, culture-dependent techniques and qPCR detected different percentages of *Starm. bacillaris*, these methods were all necessary for yeast identification and quantification analysis. Culture-dependent techniques and culture-independent techniques such as qPCR, DGGE and massive sequencing were used in this study to weigh the biases introduced by the techniques in an effort to estimate the true fungal community diversity, similarity, and composition.

### Fungal community in grape must after crushing

The main fungi in grape must from the three vineyards of the Priorat region were *Eurotiomycetes, Dothideomycetes*, and *Saccharomycetes*, all in the *Ascomycota* phylum. These fungi are commonly found in grape berries or grape must after crushing in various world wine regions (Bokulich et al., 2014; David et al., 2014; Taylor et al., 2014). The dominant species in a single vineyard were *Asper. tubingensis* (Grenache from Poboleda), *Aureo. pullulans* (Carignan from Escaladei), and *Starm. bacillaris* (Grenache from Porrera). None of these three species are plant pathogens. The high population of *Starm. bacillaris* in grape must after crushing is unexpected but understandable: approximately 31% of *Candida* (previous denomination of *Starm. bacillaris*) was found in Chardonnay grapes of Burgundy (France) (David et al., 2014), indicating the possibility of dominance of this yeast over other fungi in grape must. Moreover, some species that are considered common plant pathogens, such as *Alternaria alternate, Aspergillus niger, B. dothidea, Cladosporium cladosporioides*, and *Cytospora sacculus*, were found in low percentages (0.1–5% according to massive sequencing results). Only one sequence of *Botrytis cinerea* was found in Carignan from the Escaladei vineyard and Grenache from Porrera. No other common grape pathogen was detected. As noted by Taylor et al. (2014), the presence of DNA from these species does not necessarily mean that the grapes or plants have an infection. Fungal diseases are rare in the Priorat region because of the high temperature and low level of rainfall in the summer (Robinson, 2006). Some reads of *S. cerevisiae* (1.03%) were found in Carignan from the Escaladei vineyard but did not appear in the other two grape must samples. The low or absent evidence of DNA from *Saccharomyces* was consistent with other reports based on high-throughput sequence analysis, and with the presence of other non-dominant non-*Saccharomyces* yeasts such as *Hanseniaspora, Issatchenkia*, or *Pichia* in this study (Bokulich et al., 2014; David et al., 2014; Taylor et al., 2014).

Regional microbial “*terroir*” was proposed by Bokulich et al. (2014) as a probable explanation for the regional characteristics of final wine quality, as the fungal community was more resistant to vintage variation than regional or even vineyard variation. Our results also showed that the fungal community was more affected by geographical location than by grape variety, even though the three vineyards were all located in the Priorat region with similar altitudes and were geographically close (approximately from 5 to 12 km to each other). Interestingly, Torija et al. (2001) found that *Candida stellata* (currently renamed *Starm. bacillaris*) was the only species isolated from grape must at the same location (Porrera) in 1996. Nevertheless, the formation of grape-surface communities by vineyard or region needed more proof to be established. Furthermore, the fungal community analysis in grape must after crushing was more reliable when estimated by massive sequencing than other techniques used in this study because of the “deep community sequencing” due to the larger number of sequences analyzed (Taylor et al., 2014).

### Fungal community in grape must during wine fermentation

Fungal community dynamics during wine fermentation involve the decline of non-yeast fungi during the first 24 h, the simultaneous increase of *Hanseniaspora* species and the increase of *S. cerevisiae* at the end of fermentation. The non-yeast fungi seemed to be less tolerant of environmental change from grape skin to grape must, as few sequences were detected in grape must at 24 h, and only one sequence of *Aspergillus niger* was found in grape must at 48 h. The massive decline in non-yeast fungi contributed directly to the decreased biodiversity in grape must during fermentation. Although, the lack of detection of non-yeast fungi in grape must after 48 h resulted partly from their reduction in grape must after crushing, the decrease in non-yeast fungi could also be correlated with the dominance of *Hanseniaspora* species. A clear increase in *S. cerevisiae* appeared at the end of fermentation, which was expected (Ribéreau-Gayon et al., 2006) and was consistent in all the results with all the techniques used in this study. Only one sequence of *S. cerevisiae* was occasionally detected in grape must at 24 h, which is also consistent with the consolidated knowledge. This low percentage of *Saccharomyces* species was also observed by David et al. (2014), and in their studies, when fermentations had reached two-thirds of the process (late stages), *Saccharomyces* species were detected at lower levels. The high representation of non-*Saccharomyces* yeast in grape must (*Starmerella* in this study, and *Candida* in David et al., 2014, which could be equivalent) can account for this late detection of *S. cerevisiae* as a main species during fermentation. This competition between *Starmerella*/*Candida* and *Saccharomyces* needs further investigation.

Regardless of regional and varietal factors, fungal diversity decreased as fermentation proceeded, with the disappearance of non-yeast fungi and the predominance of non-*Saccharomyces* yeast (*Hanseniaspora*). Thus, the grape must changes during wine fermentation also seemed to affect the fungal community. However, the analysis of similarity during wine fermentation showed a high value, likely resulting from the dominance of *Starm. bacillaris* throughout the process. Moreover, the influence of SO_2_ did not change the community similarity and composition. This result was consistent with the conclusions from former studies based on culture-dependent and culture-independent techniques (Andorrà et al., 2008; Wang and Liu, 2013). However, more studies are necessary to explain how the fungal community is formed in the vineyard, the changes during wine fermentation, and the relationship between the fungal communities and regional wine characteristics.

The results from different techniques were more comparable during fermentation than in grape must. Massive sequencing was still the most comprehensive technique used in this study, as the detection of fungi is based on few sequences. For these results from massive sequencing analysis, it was important to accurately compare and search for information in the appropriate databases. To analyze the fungal community in this study, primers targeting the D1/D2 region of 26S rDNA were used due to lower differences in the sequence length and more comprehensive reference databases than for the ITS region (Taylor et al., 2014). Some other authors used different approaches based on massive sequencing: Pinto et al. (2014) analyzed sequences from both regions (D1/D2 region of 26S rDNA and ITS) to analyze the whole community, and the results indicated some variations but no significant differences were found. David et al. (2014) used amplicons of 18S rDNA for yeast diversity analysis, and the yeast dynamics trend was basically consistent with our study here. Bokulich and Mills (2013) analyzed very short amplicons from the ITS region to improve the accuracy of high-throughput sequencing, and this approach decreased the bias caused by the differences in length of conventional ITS amplicons. The amplification of different regions might provide results with fewer biases, but databases for corresponding identification are also essential if taxonomy-dependent methods are used. RDP, SILVA, and GenBank were used to assign an identity to all the sequences here (data not shown), and GenBank provided the most complete databases, with which identification at a lower taxonomical level (species) with a high confidence value of identity was achieved (Taylor et al., 2014).

In conclusion, this work indicated different fungal community diversities in grape must after crushing Grenache or Carignan grapes from three vineyards in the Priorat region of Spain. The massive sequencing analysis of grape must could provide information on the presence of plant pathogens and the species able to successfully ferment grape must. The community dynamics during wine fermentation as analyzed by qPCR, DGGE and massive sequencing showed consistent results, especially for detecting non-culturable yeast at the end of fermentation. The population changes from grape skin to grape must are related with the presence of non-*Saccharomyces* yeast on the grapes. The changes during fermentation including ethanol, nutrition, or even some yeast metabolites, introduce the appropriate conditions for the imposition of *S. cerevisiae*, which conducts the final part of the alcoholic fermentation.

## Author contributions

Conceived and designed the experiments: AM, BE. Performed the experiments: CW, DG. Generated and analyzed the data: CW, DG, AM, BE. Wrote the paper: CW, AM, BE.

### Conflict of interest statement

The authors declare that the research was conducted in the absence of any commercial or financial relationships that could be construed as a potential conflict of interest.
